# Prise en charge du COVID au Centre hospitalier Régional de Lambaréné (Gabon) de mars 2020 à février 2021

**DOI:** 10.48327/mtsi.v2i3.2022.268

**Published:** 2022-08-19

**Authors:** Madiou DIALLO, Dieudonné EYAMAMÉ

**Affiliations:** Centre hospitalier régional Georges Rawiri de Lambaréné, Gabon

**Keywords:** COVID-19, Hôpital, Traitement, Lambaréné, Gabon, Afrique subsaharienne, COVID-19, Hospital, Treatment, Lambaréné, Gabon, Sub-Saharan Africa

## Abstract

Les auteurs relatent leur expérience de prise en charge du COVID-19 au Centre hospitalier régional de Lambaréné, capitale de la province du Moyen-Ogooué au centre du Gabon. Le service de maladies infectieuses était le site de référence et de suivi pour le COVID-19, avec une équipe d'intervention pour suivre les patients non hospitalisés.

Sur 1 an en 2020, 495 cas (RT-PCR +) ont été recrutés; 92 ont été hospitalisés (comorbidités ou signes sévères).

Ces 92 cas se composent de 38 cas bénins tous guéris par traitements symptomatiques; 32 cas modérés traités et guéris; et 26 cas sévères traités par azithromycine et un 2e antibiotique avec 24 guérisons sans séquelles, 1 avec séquelles et 1 décès (co-infecté par VIH).

Les 399 cas suivis en ambulatoire ont tous guéri et se répartissent ainsi: 199 asymptomatiques et sans comorbidité, non traités; 102 avec signes bénins, sous vitamines; 98 avec signes modérés, traités par azithromycine + HCHQ + vitamines.

## Abstract (en)

The authors report on their experience of managing COVID-19 at the Regional hospital of Lambaréné, capital of the Moyen-Ogooué province in central Gabon.

The infectious diseases department was the referral and follow-up site for COVID-19, with an intervention team to follow up outpatients. The department followed national recommendations for overall management with mild cases receiving vitamin therapy, moderate cases hydroxychloroquine and azithromycin, severe cases azithromycin, another antibiotic and oxygen.

Over 1 year in 2020, 495 cases (RT-PCR +) were recruited; 92 were hospitalized (comorbidities or severe signs). The average duration of hospitalization was 21 +/- 3 days.

These 92 cases are composed of 38 mild cases all cured by symptomatic treatments; 32 moderate cases treated and cured; and 26 severe cases treated with azithromycin plus a second antibiotic with 24 cured without sequelae, 1 with sequelae and 1 death (co-infected with HIV).

The 399 cases followed up in the outpatient department all recovered and are distributed as follows: 199 asymptomatic and without co-morbidity, untreated; 102 with mild signs under vitamins; 98 with moderate signs treated with azithromycin + HCHQ + vitamins.

In total, all 495 cases recovered without sequelae except for 1 patient with sequelae and 1 death; 199 received no treatment. However, 6 deaths occurred before the diagnosis was made. This strategy encountered difficulties in terms of overloading the carers and feeding the patients.

## Introduction

Dès l'apparition des premiers cas de COVID à Libreville au Gabon, en mars 2020, la province du Moyen-Ogooué, par sa situation géographique au centre du Gabon, a été concernée. C'est ainsi que sous la présidence du Gouverneur de province, a été créé un Comité provincial du plan de veille et de riposte contre l’épidémie à Coronavirus.

Au niveau national, le premier cas a été déclaré le 12 mars 2020 et le 21 juillet les autorités nationales donnaient les chiffres suivants: 59 671 prélèvements, 6 433 cas confirmés, 4 034 guérisons et 46 décès.

## Méthodes

Le cadre de l’étude est la Région sanitaire Centre (province du Moyen-Ogooué) qui compte 69 287 habitants.

Le service de Maladies tropicales situé au Centre hospitalier régional Georges Rawiri de Lambaréné (CHRGRL), structure de référence de la Région sanitaire, a été choisi pour abriter le site de prise en charge hospitalière COVID-19 (SICOV). Ce service comporte 19 lits dont 2 réservés pour les soins intensifs.

La prise en charge des patients COVID hospitalisés a été assurée par une équipe composée de 1 médecin, 10 infirmiers, 5 psychologues, 1 hygiéniste et 2 techniciennes de surface.

Par ailleurs, les patients non hospitalisés étaient suivis par une équipe mobile EIR (Équipe d'intervention rapide) composée de 6 personnes: 1 médecin, 1 psychologue, 1 épidémiologiste, 1 technicien de laboratoire, 1 hygiéniste et 1 ambulancier. Il y a 5 équipes qui assurent chacune deux jours de garde.

Les recommandations nationales pour la prise en charge étaient les suivantes:

- Type 1 (signes bénins): azithromycine + hydroxychloroquine (HCHQ) + vitaminothérapie;
- Type 2 (signes modérés): azithromycine + amoxicilline/acide clavulanique + vitaminothérapie;
- Type 3 (signes sévères): azithromycine + céfixime + vitaminothérapie + oxygénothérapie;
- Tous les autres: traitements symptomatiques, vitaminothérapie.

## Résultats

**Figure 1:**
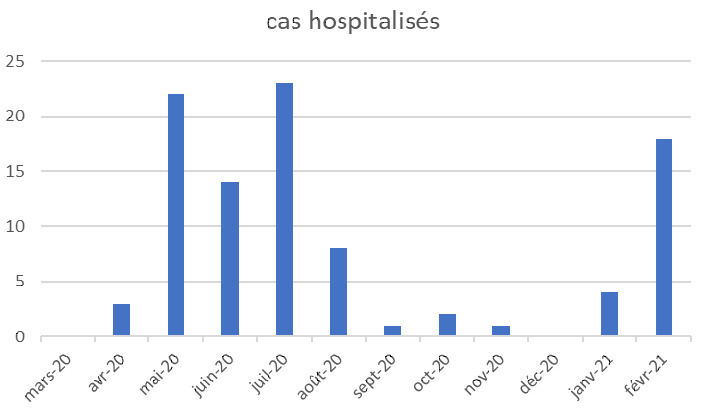
Courbe épidémique Epidemic curve

Les résultats concernent une période de 12 mois allant de mars 2020 à février 2021 (Fig. 1).

Sont inclus tous les sujets ayant eu une RTPCR positive.

Nous avons ainsi recruté 495 cas, auxquels il serait possible d'ajouter 6 cas suspects décédés avant que soit pratiqué un test PCR.

### Patients hospitalisés

Sur un total de 495 cas positifs, nous avons hospitalisé 96 patients.

Les critères d'hospitalisation étaient: comorbidités et/ou signes modérés et sévères (détresse respiratoire, altération de l’état général, syndrome infectieux)

**Figure 2:**
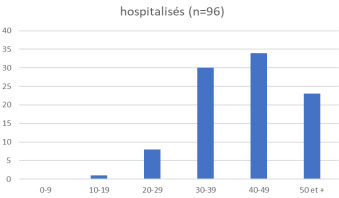
Répartition par âge Age distribution

La tranche d’âge des 40 à 49 ans représente 34,95% des cas (Fig. 2).

Le sexe-ratio H/F est de 3,5 (75 hommes; 21 femmes).

Les principales comorbidités sont les suivantes: HTA (16 cas), diabète (5 cas), HTA + diabète (4 cas), drépanocytose (3 cas), maladies respiratoires chroniques (2 cas), Sida (2 cas), et autres (2 cas).

### Résultats thérapeutiques

Sur les 96 cas hospitalisés, la durée moyenne d'hospitalisation est de 21 jours. Les critères de guérison sont constitués de deux tests PCR négatifs successifs avant la sortie de l'hôpital.

Les cas bénins au nombre de 38 ont bénéficié de traitements symptomatiques; ils ont tous guéri sans séquelles.

Les cas modérés au nombre de 32 ont été traités avec azithromycine + HCHQ + vitamines; ils ont tous guéri en 8 jours en moyenne. Sur les 32 cas, 5 n'ont pas eu de HCHQ et il n'est pas possible de mettre en évidence une différence significative entre les patients qui ont reçu de l'HCHQ et les autres (p = 0,3).

Les cas sévères, au nombre de 26, ont été traités soit avec azithromycine + amoxicilline/acide clavulanique (n = 18), soit avec azithromycine + céfixime + vitamines (n = 8). Nous avons enregistré 24 guérisons sans séquelles, 1 avec séquelles, et 1 décès (co-infection VIH). Il n'est pas possible de mettre en évidence une différence significative entre les patients ayant reçu comme deuxième antibiotique l'amoxicilline/acide clavulanique et ceux avec céfixime (p = 0,3).

Au total, le bilan est: guérison confirmée chez 88 patients (dont 1 avec séquelles), sortie avant guérison et poursuite du confinement à domicile pour 7 patients pour raisons familiales et 1 décès avec VIH+/choc septique/insuffisance respiratoire.

### Patients non hospitalisés

Les 399 patients non hospitalisés ont été suivis par une Équipe d'intervention rapide (EIR) composée de 1 médecin, 1 psychologue, 1 épidémiologiste et 1 hygiéniste.

Ce groupe comprenait: 199 asymptomatiques et sans comorbidité, non traités; 102 avec signes bénins, sous vitamines; 98 avec signes modérés, traités par azithromycine + HCHQ + vitamines.

Tous ont guéri sans séquelles.

## Discussion

Cette étude montre une bonne prise en charge globale ayant nécessité des moyens humains et matériels supplémentaires importants. Le fait que 6 patients suspects soient décédés avant la réalisation du test PCR relativise ce bon résultat et confirme par ailleurs l'importance d'une prise en charge rapide; il est néanmoins possible que ces patients aient souffert d'une autre pathologie.

L'investissement humain et matériel consacré au COVID a créé des effets collatéraux négatifs: les activités de consultations externes et d'astreintes aux urgences ont souffert de l'effort consenti. Cette situation a été déplorée au niveau national; lors de la Journée internationale de lutte contre le paludisme célébrée chaque 25 avril, le directeur du Programme national de lutte contre le paludisme au Gabon a regretté que cette nouvelle maladie ait monopolisé toutes les ressources du système de santé alors que le paludisme est toujours présent et que 15% des enfants de moins de 5 ans meurent de paludisme chaque année [2]. Un constat similaire a été fait à Abidjan avec une baisse des activités vaccinales de 45 à 90% [3]. Par ailleurs, le Fonds mondial donne des chiffres inquiétants: durant la période d'avril à septembre 2020, en comparaison avec la même période de 6 mois en 2019 le dépistage du VIH a chuté de 41%; l'orientation des cas de tuberculose a chuté de 59%; les diagnostics du paludisme ont chuté de 31%; et le nombre de consultations prénatales a chuté de 43% [1].

## Conclusion

Les principaux problèmes rencontrés dans cette prise en charge du COVID sont: le déni de la maladie par les patients et leurs familles, la stigmatisation, la durée du séjour à l'hôpital, l'absence de tomodensitométrie, le manque de réanimateurs, quelques ruptures d’équipements de protection individuelle et intrants, et parfois le burnout du personnel soignant.

Enfin, la prise en charge nutritionnelle des patients COVID est difficile à cause des moyens limités mis à disposition, qui sont devenus rares du fait de la crise. La fermeture des frontières et le confinement de la population ont engendré une pénurie alimentaire, et les marchés de Libreville font face à une pénurie de manioc, aliment de base de la population gabonaise. En conséquence, les prix ont augmenté: le bâton de manioc est passé par exemple de 300 à 400 francs CFA sur les marchés de Libreville [2].

## Liens D'intérêts

Les auteurs ne déclarent aucun lien d'intérêt.

## Contribution des Auteurs

Le Dr Eyamané est le Directeur médical de l'hôpital, il a suivi la prise en charge et il coordonnait également la gestion du materiel et le suivi des données; il a supervisé l'article

## References

[1] Le Fonds mondial (2021). Impact du COVID-19 sur les services de lutte contre le VIH, la tuberculose et le paludisme et les systèmes de santé: aperçu de la situation dans 502 établissements de santé en Afrique et en Asie. Français et anglais.

[2] Makita-Ikouaya E (2020). Le Gabon face à la COVID-19: mesures sanitaires et conséquences socio-économiques. Revue du Rhin supérieur, Centre de recherche sur les économies, les sociétés, les arts et les techniques. (CRESAT), Université de Haute-Alsace.

[3] Touré HA, Noufe S, Oussou KR, N'Guessan K, Setchi SM, Ano AMN, Tiembre I, Bénie BVJ (2021). Effets de la Pandémie à Covid-19 sur les Activités Vaccinales D'un Centre de Vaccination de Référence de Treichville en Côte D'ivoire [Effects of Covid-19 Pandemic on the Vaccine Activities on a Reference Immunization Center in Treichville, Côte D'ivoire]. Med Trop Sante Int.

